# Characterization of endovascular rescue of massive gastroduodenal artery hemorrhage following upper gastrointestinal surgery

**DOI:** 10.3389/fmedt.2026.1746432

**Published:** 2026-04-10

**Authors:** Zhiyuan Zhou, Lei Zheng, Weiqing Tang, Bin Liu, Pengfei Wang, Chaoran Yu, Weimin Wang

**Affiliations:** 1 Department of General Surgery, Shanghai Ninth People's Hospital, Shanghai Jiao Tong University School of Medicine, Shanghai, China; 2 Department of Interventional Radiology, Shanghai Ninth People's Hospital, Shanghai Jiao Tong University School of Medicine, Shanghai, China; 3 Division for Shanghai Ninth People's Hospital, China Hospital Development Institute, Shanghai Jiao Tong University, Shanghai, China

**Keywords:** arterial embolization, emergent operation, gastroduodenal artery bleeding, massive arterial hemorrhage, postoperative

## Abstract

**Background:**

Postoperative hemorrhage from the gastroduodenal artery (GDA) is a rare but life-threatening complication following upper gastrointestinal surgery. Its management is particularly challenging due to the frequent co-occurrence of hypovolemic shock, complex intra-abdominal infection, and malnutrition, which collectively contribute to high morbidity and mortality.

**Methods:**

We conducted a retrospective analysis of patients who underwent either gastrectomy or pancreaticoduodenectomy and subsequently developed delayed massive GDA bleeding between January 2021 and June 2025 in the Department of General Surgery, Shanghai Ninth People's Hospital, Shanghai Jiao Tong University School of Medicine.

**Results:**

Six patients was included in this study, of whom five had undergone radical D2 lymphadenectomy and developed duodenal stump leakage or anastomotic leakage and one had undergone pancreaticoduodenectomy. All cases experienced massive postoperative arterial hemorrhage. Emergent angiographic arterial embolization was successfully performed in all cases, achieving effective hemostasis with satisfactory outcomes. Specifically, one patient required four sequential interventions, one required three, one required two, and the remaining three achieved hemostasis after a single emergent procedure. Notably, acute liver function abnormalities were observed in two cases following embolization of the common hepatic artery.

**Conclusion:**

Massive postoperative GDA hemorrhage is a life-threatening complication after abdominal surgery. Emergent arterial embolization proves to be a rapid, minimally invasive, effective, and safe therapeutic option for this critical condition.

## Introduction

A considerable proportion of patients undergoing upper gastrointestinal surgery experience at least one postoperative complication within 90 days, with up to 32.3% of these cases classed as Clavien–Dindo Grade III/IV (1–3). Other studies have reported a 30-day morbidity of 23.6% and a 30-day mortality ranging from 2.0% to 4.1% (1, 2). The occurrence of postoperative complications is influenced by numerous factors, including surgeon experience, patient age, and quality of surgical training (4–6). While surgical techniques for upper gastrointestinal procedures continue to evolve, the investigation of postoperative complications—particularly catastrophic events such as massive gastroduodenal artery (GDA) hemorrhage which carries an extremely high mortality rate—appears to receive less attention in the literature (7).

A 10-year retrospective review of gastrointestinal leakage in Korean gastric cancer patients identified three Clavien–Dindo Grade V and two Grade IVb cases among 198 patients with postoperative leakage (8, 9). Postoperative massive arterial hemorrhage from GDA bleeding is undeniably one of the most dangerous and fatal complications, characterized by a rapid clinical course and formidable therapeutic challenges (10–13). Non-operative or conventional management options are often ineffective due to the presence of concurrent malnutrition, complex intra-abdominal infection, and limited surgical experience in managing such acute scenarios. In this report, we present our experience with a safe and rapid endovascular intervention for massive postoperative GDA bleeding. We describe a series of six patients with Grade IVb complications managed successfully over the past 4 years, all of whom achieved satisfactory hemostasis following emergent arterial embolization.

## Materials and methods

A total of six patients were included in this study, comprising five who underwent radical D2 lymphadenectomy and one who underwent pancreaticoduodenectomy in the Department of General Surgery, Shanghai Ninth People's Hospital, between January 2021 and June 2025. Inclusion criteria consist of primary D2 lymphadenectomy or pancreaticoduodenectomy, postoperative massive abdominal bleeding, and emergent angiographic embolization. All patients who developed delayed massive bleeding during their postoperative hospital stay were confirmed to have GDA bleeding. This study was a retrospective study. Clinical data and therapeutic strategies were retrospectively collected and analyzed, including demographics, pathological findings, comorbidities, complication types, clinical outcomes, and details of emergency angiographic embolization. The follow-up duration was 30 days after hospital discharge.

Prior to emergency arterial embolization, all patients received standard clinical therapies, including volume resuscitation, correction of coagulopathy, administration of proton pump inhibitors, placement of double-lumen irrigation-suction tubes, and endoscopic interventions. One patient additionally underwent emergent open surgery prior to embolization. Angiographic arterial embolization was performed using standard percutaneous transfemoral catheterization. Selective opacification of the GDA and common hepatic artery (CHA) was achieved, and embolization was performed using vascular coils and microcoils targeting the identified bleeding vessels.

## Results

### Emergent arterial embolization

**Figure 1:**
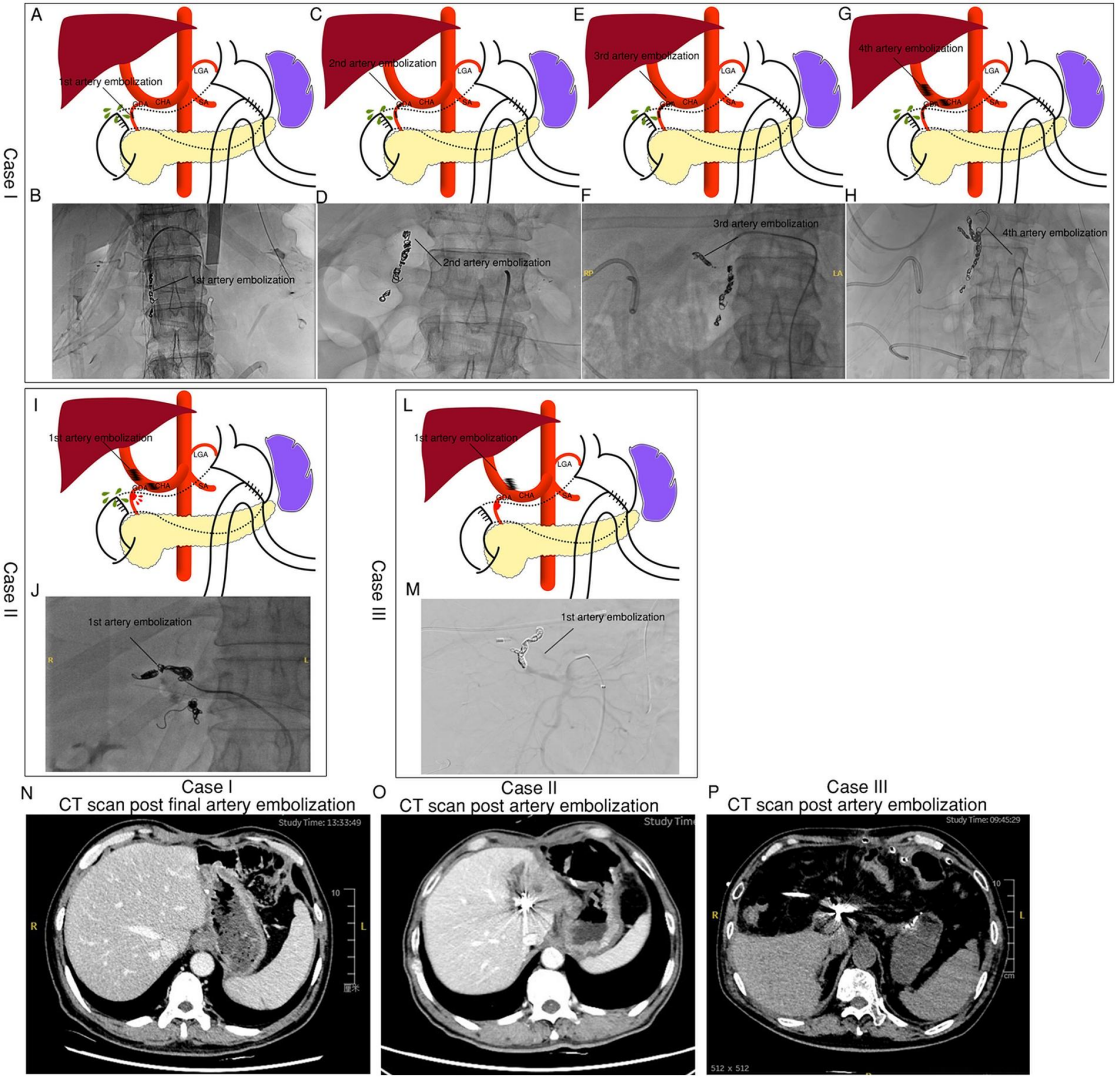
Demonstration of two gastric cancer cases with radical D2 lymphadenectomy and Billroth II, which were diagnosed as duodenal stump leakage and delayed massive artery hemorrhage, followed by emergent artery embolization of the common hepatic artery, proper hepatic artery, and gastroduodenal artery. **(A)** Demonstration of artery embolization and duodenal stump leakage site in Case I; **(B)** artery hemorrhage and embolization of first DSA in Case I; **(C,D)** second artery hemorrhage and embolization of DSA in Case I; **(E,F)** third artery hemorrhage and embolization of DSA in Case I; **(G,H)** fourth artery hemorrhage and embolization of DSA in Case I; **(I,J)** artery hemorrhage and embolization of DSA in Case II; **(L,M)** artery hemorrhage and embolization of DSA in Case III; **(N–P)** DSA postoperative CT scan post-final artery embolization in Cases I **(N)**, II **(O)**, and III **(P)**.

Of the six patients, three who underwent radical D2 lymphadenectomy with Billroth II reconstruction developed duodenal stump leakage followed by delayed massive arterial hemorrhage (Figure 1, Tables 1 and 2).

**Table 1:** Clinical characterization of six patients received both gastrectomy and emergency artery embolization.

Patient information	Case I	Case II	Case III	Case IV	Case V	Case VI
Gender	Male	Male	Male	Male	Male	Female
Age	51	52	74	59	80	58
Primary lesion	Gastric pyloric antrum	Gastric angle	Gastric pyloric antrum	Gastric angle	Gastric pyloric antrum	Periampullary Vater
Diameter of lesion		2 cm × 2 cm	4 × 5	2.5 cm × 2.2 cm		
Major complains	Digestive bleeding	Health screen	Digestive bleeding	Abdominal bloating	Digestive bleeding	Health screen
CA50 U/mL	11.73	97.32	<1	—	50	—
CA242 U/mL	10.22	43.83	<1	—	38.42	—
CA19-9 U/mL	13.3	154	2	6.21	51.5	619
CA125 U/mL	7.11	13	9.1	7.48	32.9	12.7
Alpha Fetoprotein (AFP) IU/mL	1.49	1.65	1.24	1.45	0.75	1.5
Carcinoembryonic antigen (CEA) ng/mL	1.53	57.4	2.1	2.86	12.8	1.68
HB AT ADMISSION g/L	152	149	130	138	65	146
Initial operations	Billroth II + distal gastrectomy	Billroth II + distal gastrectomy + microwave ablation	Billroth II + distal gastrectomy	Billroth I + distal gastrectomy	Billroth I + distal gastrectomy	Pancreaticoduodenectomy
Postoperative complications	GDA bleeding	GDA bleeding and delayed pseudoaneurysm	GDA bleeding	GDA bleeding	GDA bleeding	GDA bleeding
Emergency DSA intervention	Four DSA emergency operations	One DSA emergency operation	One DSA emergency operation	Three DSA emergency operations	One DSA emergency operation	Two DSA emergency operations

**Table 2:** Postoperative pathological characterizations of six patients.

Patient data	Case I	Case II	Case III	Case IV	Case V	Case VI
Pathology T	T3	T4	T4	T1	T3	T1
Pathology N	N1	N3	N0	N0	N1	N1
Pathology M	M0	M1	M0	M0	M0	M0
Positive LN	1	11	0	0	1	1
Station of positive LN	Lesser curvature	Greater + lesser curvature	NA	NA	Greater curvature	superior mesenteric vein (SMV)
Total LN	15	17	17	19	15	1
Nerve invasion	+	+	+	−	+	−
Vascular invasion	+	+	+	−	+	−
Histology	Poorly differentiated, signet ring cell	Moderate differentiated adenocarcinoma	Moderate differentiated adenocarcinoma	Poorly differentiated, signet ring cell	Moderate differentiated	Highly differentiated adenocarcinoma
Isolated tumor node	−	NA	+	NA	NA	NA

In Cases I and III, the first postoperative bleeding episode occurred 9 days after surgery, presenting as sudden bloody output from abdominal drains and clinical signs of hypovolemic shock. Initial management included coagulopathy correction, endoscopic evaluation, and volume resuscitation, followed by emergent angiographic arterial embolization (Table 3). Active GDA bleeding and duodenal stump leakage were confirmed. The GDA terminus was successfully embolized. However, Case I experienced recurrent bleeding requiring a second embolization 19 days postoperatively, with the embolization point proximal to the initial site. A third embolization was performed at day 29, targeting the GDA root and several terminal branches originating from the superior mesenteric artery. Three days later, the patient developed massive gastrointestinal bleeding (hematemesis and melena), necessitating a fourth embolization involving the common and proper hepatic arteries (Table 3). Following intensive support care, this patient was discharged after a 77-day hospital stay.

**Table 3:** Emergency DSA operations schema of six patients.

DSA sequence	Case I	Case II	Case III	Case IV	Case V	Case VI
First DSA	Postoperative day (POD) 9 GDA embolization at the end of the ligation point	POD 16 GDA and common hepatic artery and proper hepatic artery embolization	POD 9 GDA embolization	POD 21 GDA embolization	POD 26 GDA embolization	POD 20 GDA and right hepatic artery embolization
Second DSA	POD 19 GDA embolization at the proximal of the last embolization point	—	—	POD 40 GDA embolization	—	POD 21 GDA embolization
Third DSA	POD 29 GDA embolization at the root of GDA and other branches of SMA	—	—	POD 68 GDA embolization and branches of superior mesenteric artery (SMA)	—	—
Fourth DSA	POD 32 common hepatic artery and proper hepatic artery embolization	—	—	—	—	—
In-hospital stay	77 days	63 days	46 days	84 days	69 days	77 days
Outcome	Alive	Alive	Alive	Alive	Alive	Alive

Another patient in this subgroup (Case II) experienced sudden massive bleeding on postoperative day 16, evidenced by bloody drainage and hematemesis. Immediate blood transfusion and hemostatic therapy were initiated. Angiography revealed a possible pseudoaneurysm at the junction of the GDA and common hepatic artery (Table 3), prompting embolization of the proper hepatic artery (PHA), GDA, and common hepatic artery.

**Figure 2:**
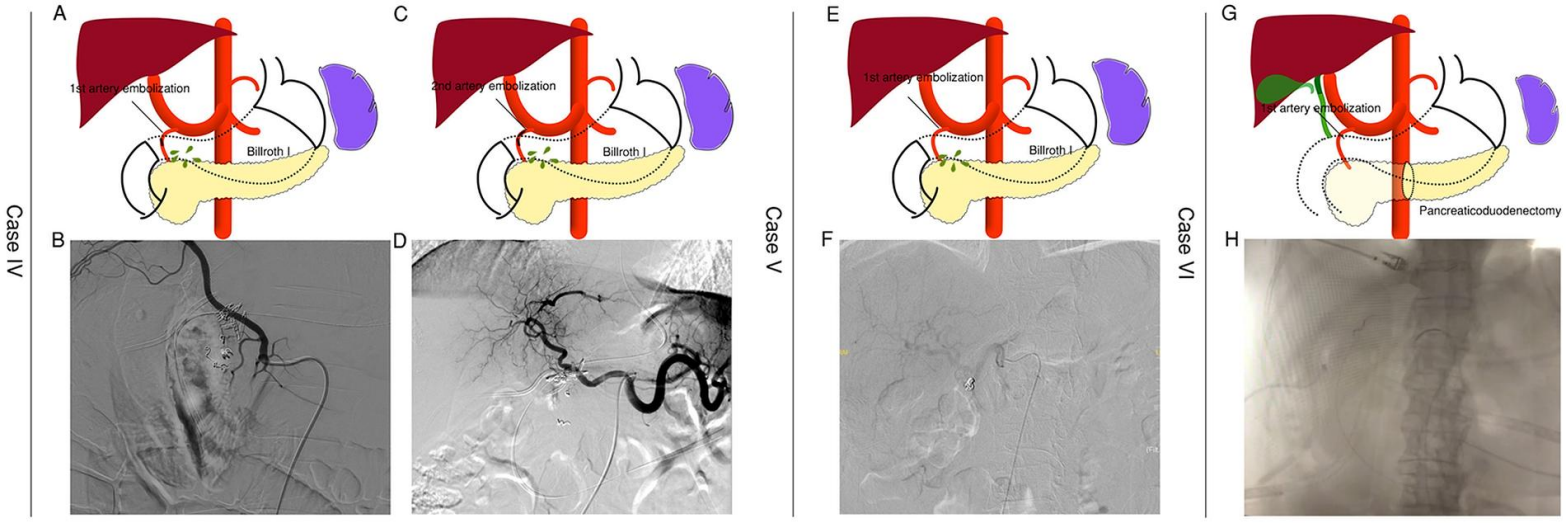
Demonstration of two gastric cancer cases with radical D2 lymphadenectomy and one case with pancreaticoduodenectomy, which were diagnosed as delayed massive artery hemorrhage, followed by emergent artery embolization of the gastroduodenal artery (GDA). **(A,B)** Demonstration of artery embolization and anastomosis leakage site and emergent artery hemorrhage in DSA in Case IV; **(C,D)** second artery embolization in Case IV; **(E,F)** demonstration of artery embolization and anastomosis leakage site and artery hemorrhage in DSA in Case V; **(G,H)** demonstration of artery embolization and DSA in Case VI.

Two patients who underwent radical D2 lymphadenectomy with Billroth I reconstruction developed anastomotic leakage followed by delayed massive arterial hemorrhage (Figure 2, Table 1). One patient presented with hematemesis and melena on postoperative day 40. Following initial fluid resuscitation, hemostatic therapy, and blood product support, the patient remained hemodynamically unstable. Emergency angiography identified active GDA bleeding, which was embolized, achieving temporary stabilization. However, 17 days later, recurrent massive hematemesis and melena required a second emergent GDA embolization.

The other patient exhibited massive bleeding from an abdominal drain on postoperative day 26, accompanied by signs of hypovolemia. Emergent angiography confirmed GDA as the bleeding source, and immediate embolization successfully stabilized the patient's vital signs (Table 3). Anastomotic leakage was also confirmed. Both patients were ultimately discharged safely.

### Abnormal liver function monitor

**Figure 3:**
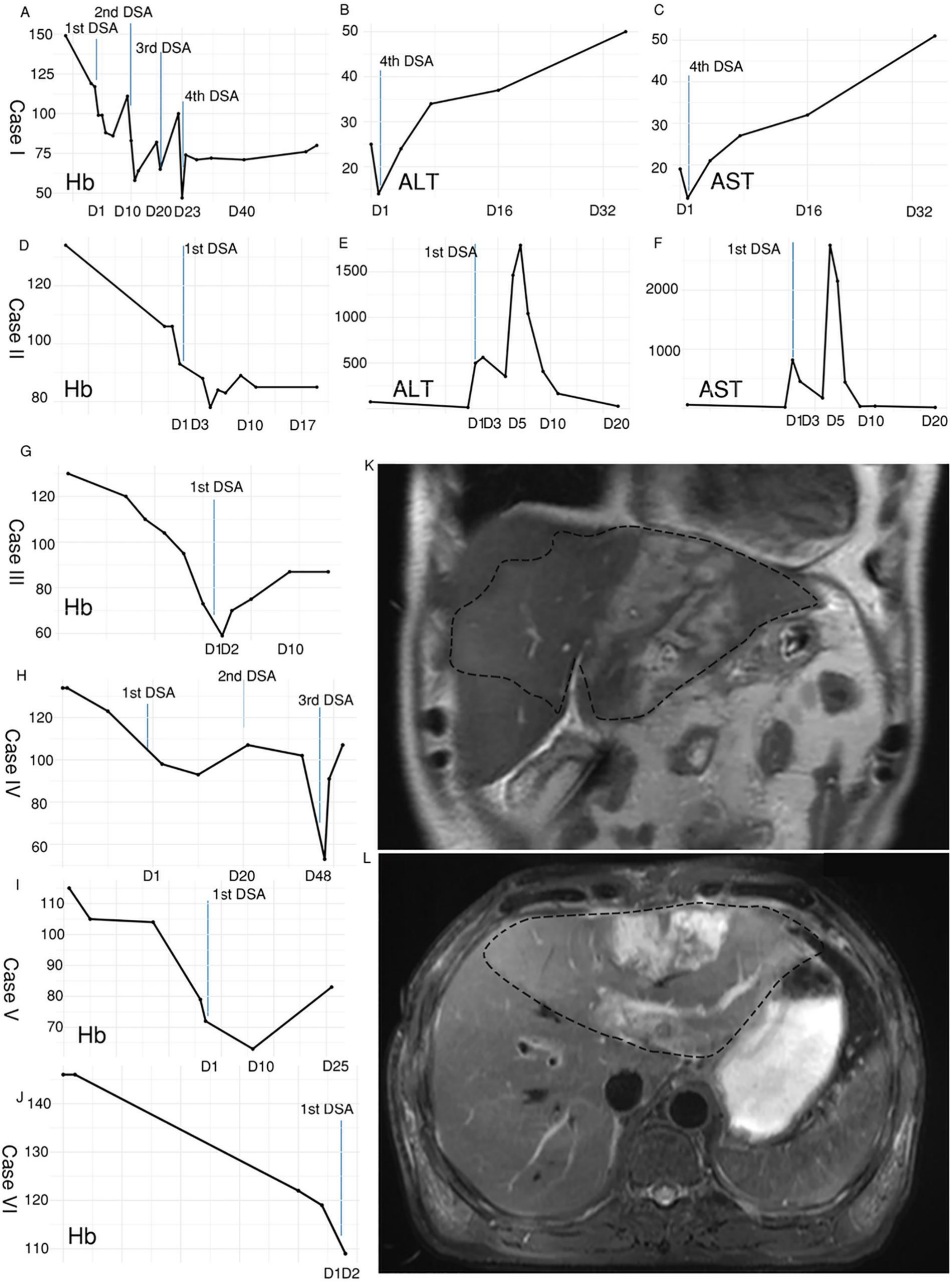
Hb levels in six cases and liver function in two cases in response to the DSA courses. **(A)** Hb level during the DSA course in Case I; **(B)** ALT level during the DSA course in Case I; **(C)** AST level during the DSA course in Case I; **(D)** Hb level during the DSA course in Case II; **(E)** ALT level during the DSA course in Case II; **(F)** AST level during the DSA course in Case II; **(G)** Hb level during the DSA course in Case III; **(H)** Hb level during the DSA course in Case IV; **(I)** Hb level during the DSA course in Case V; **(J)** Hb level during the DSA course in Case VI; **(K,L)** abnormal liver signal MRI comparison due to ischemia 1 week post DSA in Case II.

**Figure 4:**
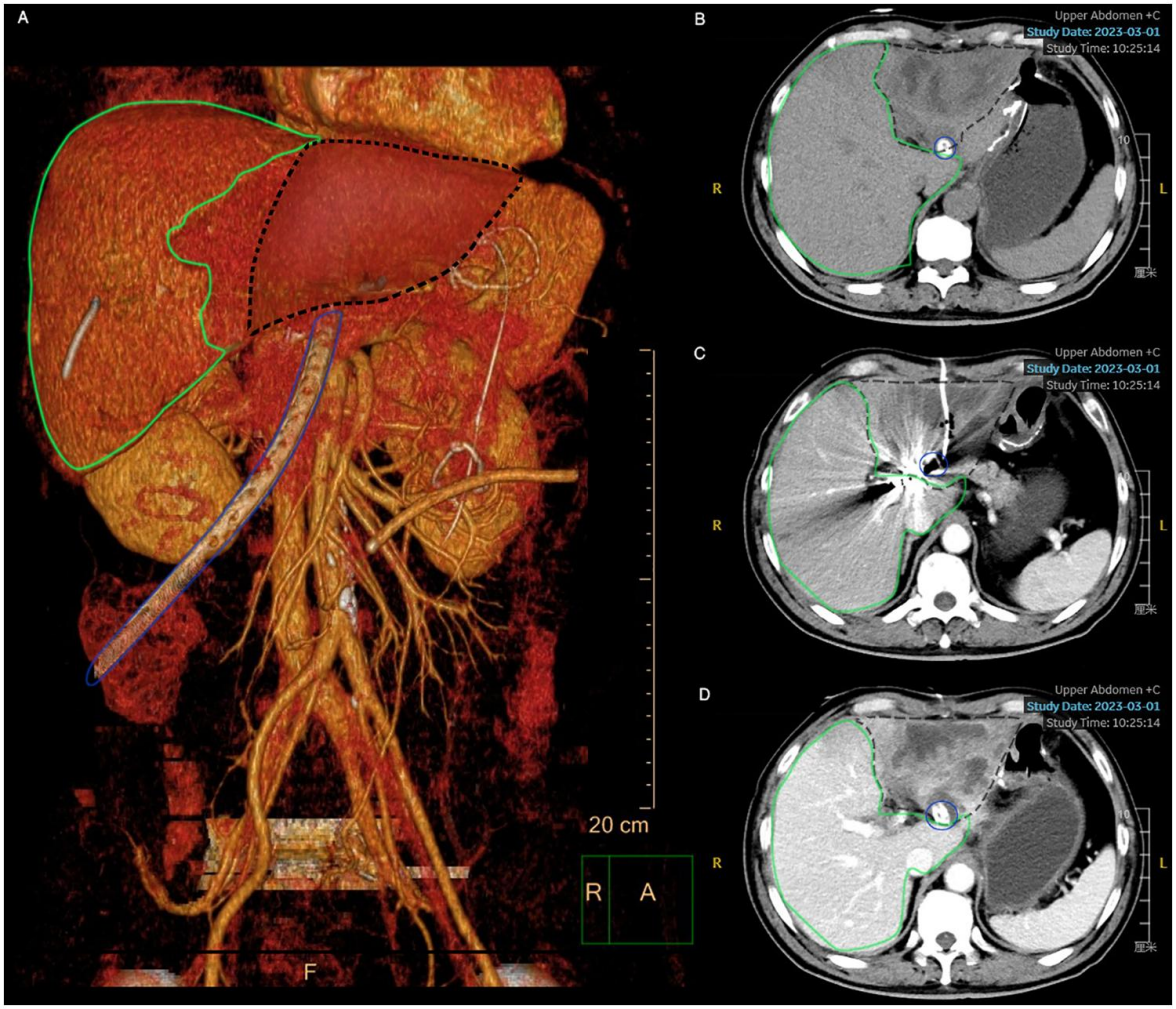
Three-dimensional contrast-enhanced CT series of liver abscess following liver ischemia post-emergent DSA in 2 weeks. **(A)** Volume rendering of liver and primary operation area; blue line indicates double-space irrigation tube; **(B–D)** contrast-enhanced CT of liver abscess in plain scan/arterial phase/portal venous phase; green line indicates normal area of liver; black dash indicates liver abscess resulting from liver ischemia post DSA with common hepatic artery and proper hepatic artery embolized.

Hemoglobin levels were monitored throughout the peri-embolization period. Notably, a marked elevation in alanine aminotransferase (ALT) and aspartate aminotransferase (AST) levels in Cases I and II indicated acute liver function impairment (Figure 3). In Case I, ALT and AST peaked at 1,790 U/L and 2,761 U/L, respectively, 4 days after arterial embolization. In Case II, an MRI performed 1 week post-embolization revealed a distinct hepatic ischemic area and developing liver abscess (Figure 3). This patient subsequently received CT-guided abdominal drainage and supportive therapy. Follow-up CT with volume rendering indicated successful disease control and a satisfactory final outcome (Figure 4). The patient was discharged with normal liver function and adequate oral intake after a 63-day hospital stay.

### Therapeutic pipeline

Based on our clinical experience, we proposed a two-stage management strategy structured according to disease progression (Figure 4). The initial phase involves early identification of signs of gastrointestinal leakage (anastomotic or duodenal stump). As the condition progresses, acute bleeding typically occurs between postoperative days 7 and 21, initiating Stage I therapy: emergent arterial embolization. This stage requires prompt diagnosis via computed tomography angiography (CTA) or digital subtraction angiography (DSA) to precisely localize the bleeding vessel, followed by superselective embolization or other non-operative interventions. Repeated embolizations may be necessary for recurrent bleeding. Notably, in high-risk situations, embolization of the common hepatic artery (CHA) and proper hepatic artery (PHA) might be unavoidable. Stage II therapy subsequently focuses on the detection and management of potential hepatic ischemia, particularly in cases where the CHA/PHA was embolized. Early recognition and intervention for liver failure or abscess are critical to optimizing patient outcomes.

## Discussion

**Figure 5:**
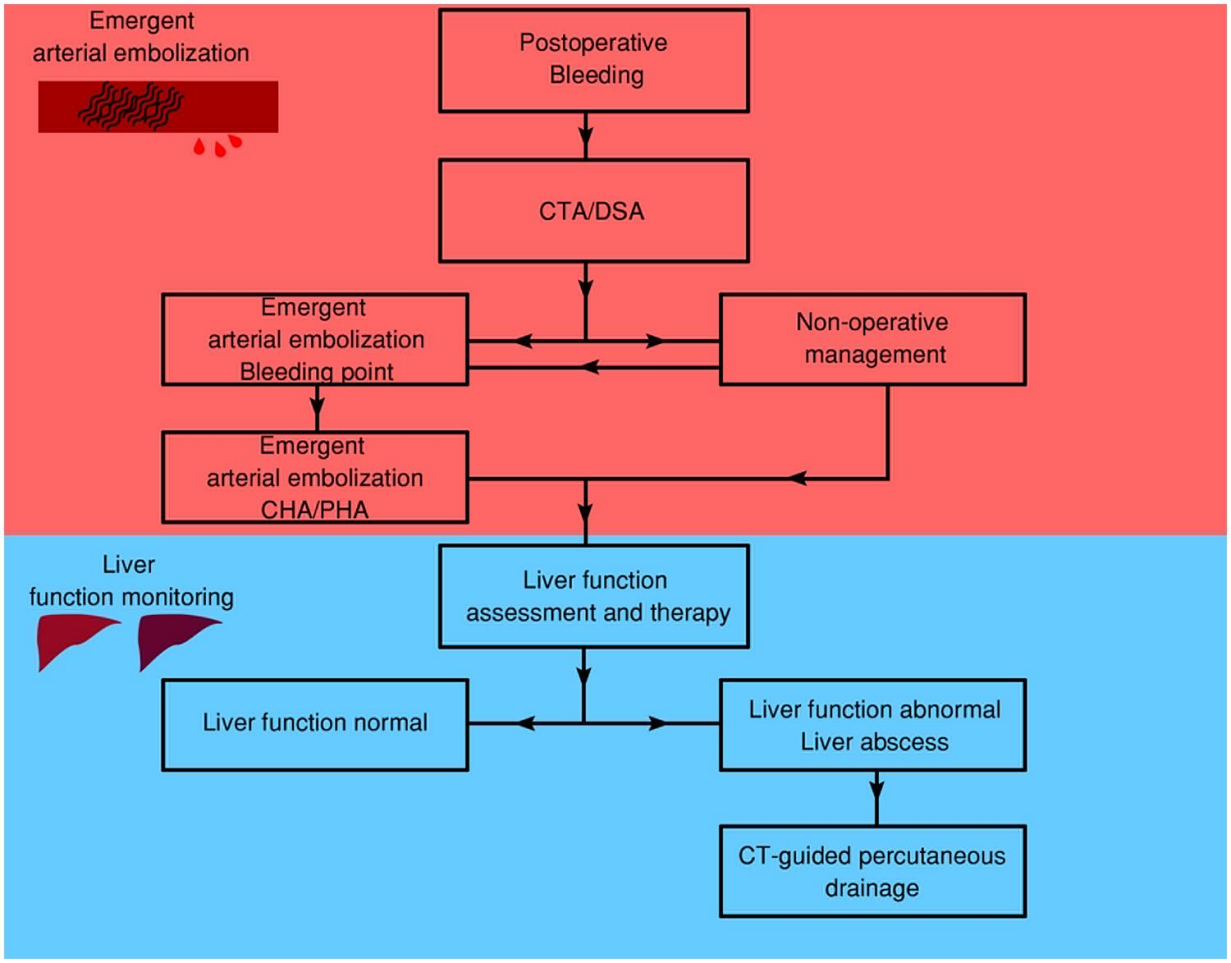
Consort diagram of clinical therapeutic management (Stage I–II) of massive postoperative gastroduodenal hemorrhage in gastrointestinal leakage after gastrectomy for gastric cancer. Stage I: emergent arterial embolization for immediate identification of abdominal bleeding. Stage II: liver function monitoring post arterial embolization (CHA/PHA). CHA, common hepatic artery; PHA, proper hepatic artery.

This study demonstrates a high technical success rate for emergent arterial embolization in managing massive postoperative GDA hemorrhage, with no 30-day mortality and effective hemostasis achieved through a two-stage therapeutic protocol (Figure 5). These outcomes compare favorably with previous studies, which reported variable success rates and mortality rates as high as 39.1% (14, 15) (Table 4).

**Table 4:** Comparison of success rate and mortality rate in studies with emergency artery embolization.

Study	Patients (*N*)	Success rate of emergent artery embolization	30-day mortality
Burris et al. (14)	8	—	12.5%
Loffroy et al. (15)	33	81.8%	21.2%
Wong et al. (16)	32	88.5%	25%
Ripoll et al. (17)	31	83.9%	25.8%
Eriksson et al. (18)	40	—	3%
Langner et al. (19)	11	—	27%
Defreyne et al. (20)	46	90%	39.1%
Larssen et al. (21)	36	92%	19%
Venclauskas et al. (22)	24	—	20.8%
Ang et al. (23)	30	80%	16.7%
Jairath et al. (24)	60	—	10%
Jailani et al. (25)	24	67%	33%
Laursen et al. (26)	45	60%	29%
Griffiths et al. (27)	24	58%	20.8%
Nykänen et al. (28)	42	75%	12.5%

### Therapeutic protocol and clinical implications

The two-stage protocol we propose addresses the clinical trajectory of these high-risk patients. The initial stage focuses on the prompt identification and management of gastrointestinal leakage and intra-abdominal infection, which are recognized as precursors to catastrophic bleeding. The second stage provides a framework for management of recurrent hemorrhage and its sequelae, particularly in monitoring and treatment of hepatic dysfunction following extensive arterial embolization.

In our series, one patient required four sequential embolizations due to recurrent hemorrhage, and two others required two procedures. These cases highlight the complexity of achieving durable hemostasis. Suboptimal success rates of arterial embolization in previous studies have been attributed to several key factors, which remain topics of debate among surgeons and interventional radiologists (13). We expand on this discussion based on our experience.

### Factors associated with embolization outcomes

First, anatomical variations and abnormalities, such as tumor-related neovascularity or atypical branching patterns of the GDA, can present case-specific challenges that complicate superselective catheterization and effective embolization. Such variations may prolong procedure duration and reduce the precision of vessel occlusion. Second, concurrent abdominal infection and gastrointestinal leakage exacerbate tissue status, increasing vessel fragility and surrounding inflammation and the risk of delayed or recurrent hemorrhage even after successful embolization, technically. Another critical factor is the timing for intervention. Accurate diagnosis of gastrointestinal hemorrhage can be challenging, and organizing salvage therapy such as transcatheter arterial embolization (TAE) requires prompt discussion or coordinating a decision by a multidisciplinary team. Delays in this process may increase mortality.

### Role of endovascular therapy in a high-risk population

Systematic management of massive postoperative arterial hemorrhage is often ineffective using conventional approaches. Given the frequent coexistence of malnutrition and complex intra-abdominal infection, emergent reoperation carries a prohibitively high mortality risk. Although endoscopic techniques have advanced, their utility in massive gastroduodenal hemorrhage remains limited. Therapeutic angiography has been recognized since the 1970s as an alternative for bleeding control, especially in high-risk surgical candidates or patients who are poor surgical candidates. Superselective transcatheter embolization has become a feasible option for both upper and lower gastrointestinal (GI) bleeding when endoscopy fails or surgery is contraindicated (11).

It is important to note that angiographic confirmation of an active bleeding site is not always a prerequisite. Previous studies note that angiographic extravasation may not be visible in all cases of massive upper GI bleeding (29), potentially due to temporary cessation of bleeding. Theoretical thresholds for angiographic detection of contrast extravasation require active bleeding at a rate of at least 0.5–1.0 mL/min (30).

Complications following arterial embolization are rare but can be serious, including hemorrhage recurrence, hepatic or gastric ischemia, gastric necrosis, and others. In this series, three patients with duodenal stump leakage underwent embolization of the CHA, PHA, and GDA. One patient demonstrated immediate elevations in ALT and AST, consistent with acute hepatic ischemia. This study also aligns with previous studies on severe hepatic trauma, reporting that primary hepatic artery embolization success rates of 80%–97%, with complications including hepatic ischemia and bilomas (31). However, patients with combined gastrointestinal leakage, intra-abdominal infection, and arterial embolization may have a higher risk of hepatic ischemia and subsequent liver failure. Indeed, a liver abscess was identified via MRI in the same patient with acute liver function abnormalities, necessitating CT-guided percutaneous drainage. Similarly, another case report described a delayed massive upper GI hemorrhage 3 months post-total gastrectomy, managed by emergent CHA embolization with microcoils and gelatin sponges, attributed to vascular erosion from chronic digestive juices and chemotherapy-induced vomiting (32).

### Role of drainage in infection control

The double-lumen irrigation-suction tube has been validated as effective for draining intra-abdominal infections (33–35). Yao et al. (33) reported superior therapeutic advantages for this device compared to secondary or delayed primary closure. Another study found it more effective than passive drainage, reducing costs and hospital stay (35). In our study, this device played a vital role in initial management upon identifying gastrointestinal leakage, supporting its efficiency in mitigating intra-abdominal infection.

### Demographic and clinical variables

Notably, four patients in this study were male, consistent with a report from Seoul National University identifying male sex as an independent risk factor for anastomotic leakage in gastric cancer (36). That study reported a leakage rate of 1.88% (72/3,827), with 19.4% at the duodenal stump, 27.8% at gastroduodenostomy, and the highest rate (36.1%) at esophagojejunostomy (36). Tumors in the upper third of the stomach were also a potential risk factor, although only 19.2% of patients had tumors in this location. The mean hospital stay in that study was 15.8 days (range: 9–33), considerably shorter than our mean of 73.3 days (range: 63–84). This difference is likely attributable to the absence of massive bleeding in their cohort and the fact that 62 of 72 leakage cases were managed conservatively without the need for invasive intervention.

Postoperative anastomotic hemorrhage remains a significant concern. Tanizawa et al. (37) reported 6 cases among 1,400 gastrectomies, with bleeding sites localized to the gastroduodenostomy in 5 cases, managed endoscopically. Another study of 2,031 gastrectomies identified 7 cases of postoperative anastomotic bleeding (38). Such bleeding often originates from anastomotic suture or staple lines, relating to instrumentation or surgical technique.

### Study limitations and future directions

The clinical management of massive postoperative GI arterial bleeding, especially when complicated by concurrent leakage, remains a major, non-standardized challenge. Emergent endovascular arterial embolization offers advantages over conventional open surgery, including the potential to reduce mortality and complications related to malnutrition and infection. As such, it has emerged as a critical alternative for high-risk surgical candidates. Acknowledgeably, this retrospective study may not establish the superiority over other potential alternative treatment modalities, but it still provides meaningful real-world clinical evidence favoring the emergent endovascular rescue strategy.

Future efforts should focus on refining embolization techniques (e.g., superselective skills and advanced embolic agents), improving early diagnosis (e.g., predictive biomarkers and AI-enhanced dynamic imaging) to facilitate more timely intervention, and reducing risks of hepatic ischemia and recurrent hemorrhage (e.g., anatomic risk assessment and multimodal organ support) which will require improved anatomical risk assessment and multiorgan support strategies.

Given the rarity of the cases, standardizing management requires more multicenter data and evidence-based protocols to minimize heterogeneity bias and establish balanced clinical algorithms. A multidisciplinary team is essential for optimizing surgical technique, postoperative care, and personalized risk mitigation.

### Technological innovations

Technological advancements continue to expand the potential of angiographic embolization. Cone-beam CT angiography (CBCTA) provides 3D vascular mapping, potentially reducing procedure time and contrast load, especially in complex cases. Novel embolic agents, such as “hourglass”-shaped devices, may offer more stable vessel occlusion and lower recanalization rates. While direct abdominal drainage may not directly cause liver abscess, its early application could potentially help prevent this complication in the long term.

## Conclusion

Massive postoperative hemorrhage from GDA bleeding is a life-threatening complication following abdominal surgery. Emergent arterial embolization represents a rapid, effective, and safe therapeutic option in these critical scenarios, despite the challenges posed by recurrent bleeding and potential hepatic complications.

## Data Availability

The raw data supporting the conclusions of this article will be made available by the authors, without undue reservation.
